# 2-Methyl­pyrazine 1,4-dioxide

**DOI:** 10.1107/S1600536809046534

**Published:** 2009-11-07

**Authors:** Jessica L. Gratton, Jacqueline M. Knaust

**Affiliations:** a Allegheny College, Chemistry Department, 520 North Main St., Meadville, PA 16335, USA

## Abstract

The title compound, C_5_H_6_N_2_O_2_, was prepared from 2-methyl­pyrazine, acetic acid and hydrogen peroxide. In the crystal, π–π stacking inter­actions between neighboring mol­ecules are observed, with a centroid–centroid distance of 3.7370 Å, an inter­planar distance of 3.167 Å, and a slippage of 1.984 Å. Each mol­ecule is linked to four neighbors through C—H⋯O hydrogen-bonding inter­actions, forming one-dimensional ribbons.

## Related literature

For the synthesis of 2,2′-bipyridine *N,N*’-dioxide, see: Simpson *et al.* (1963 ▶). For the synthesis of lanthanide coordination networks with pyrazine *N,N*’-dioxide, see: Cardoso *et al.* (2001 ▶); Sun *et al.* (2004 ▶). For the use of 2-methyl­pyrazine 1,4-dioxide in the synthesis of a cadmium (II) coordination network, see: Shi *et al.* (2006 ▶). For the use of 2-methyl­pyrazine 1,4-dioxide in the synthesis of several mol­ecular complexes, see: Sun *et al.* (2005 ▶); Xu *et al.* (2005*a* ▶,*b* ▶).
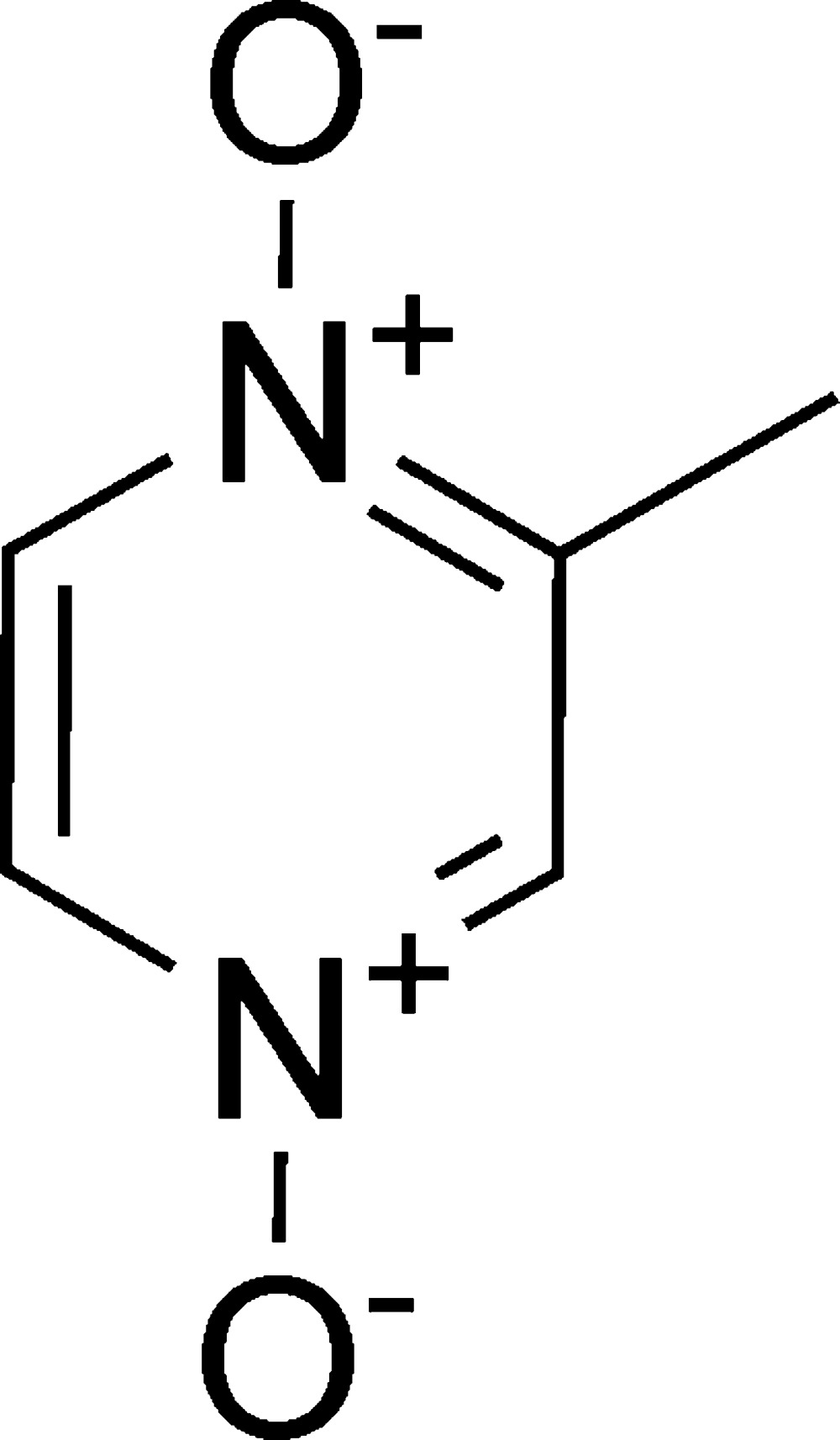


## Experimental

### 

#### Crystal data

C_5_H_6_N_2_O_2_
*M* *_r_* = 126.12
Orthorhombic, 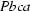

*a* = 6.3953 (9) Å
*b* = 12.2472 (18) Å
*c* = 13.6613 (19) Å
*V* = 1070.0 (3) Å^3^
*Z* = 8
Mo *K*α radiation
μ = 0.12 mm^−1^
*T* = 173 K
0.53 × 0.20 × 0.15 mm

#### Data collection

Bruker SMART APEX CCD diffractometer
Absorption correction: multi-scan (*SADABS*; Bruker, 2001 ▶) *T*_min_ = 0.763, *T*_max_ = 1.000
5556 measured reflections
1708 independent reflections
1407 reflections with *I* > 2σ(*I*)
*R*_int_ = 0.025

#### Refinement

*R*[*F* ^2^ > 2σ(*F* ^2^)] = 0.046
*wR*(*F* ^2^) = 0.122
*S* = 1.07
1708 reflections
83 parameters
H-atom parameters constrained
Δρ_max_ = 0.45 e Å^−3^
Δρ_min_ = −0.31 e Å^−3^

### 

Data collection: *SMART* (Bruker, 2007 ▶); cell refinement: *SAINT-Plus* (Bruker, 2007 ▶); data reduction: *SAINT-Plus*; program(s) used to solve structure: *SHELXS97* (Sheldrick, 2008 ▶); program(s) used to refine structure: *SHELXL97* (Sheldrick, 2008 ▶); molecular graphics: *X-SEED* (Barbour, 2001 ▶); software used to prepare material for publication: *X-SEED*.

## Supplementary Material

Crystal structure: contains datablocks I, global. DOI: 10.1107/S1600536809046534/zl2250sup1.cif

Structure factors: contains datablocks I. DOI: 10.1107/S1600536809046534/zl2250Isup2.hkl

Additional supplementary materials:  crystallographic information; 3D view; checkCIF report

## Figures and Tables

**Table 1:** Hydrogen-bond geometry (Å, °)

*D*—H⋯*A*	*D*—H	H⋯*A*	*D*⋯*A*	*D*—H⋯*A*
C1—H1⋯O2^i^	0.93	2.31	3.2224 (16)	165
C2—H2⋯O2^ii^	0.93	2.23	3.1405 (17)	167
C3—H3⋯O1^iii^	0.93	2.29	3.2090 (15)	168

Symmetry codes: (i) 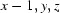; (ii) 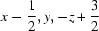; (iii) 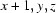.

**Fig. 1.**
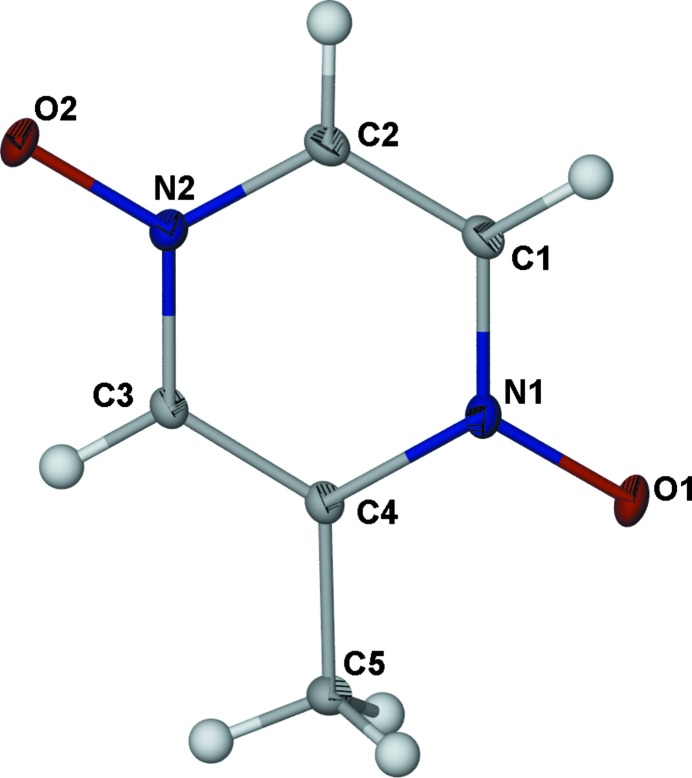
The molecular structure of the title compound with atom labels and 50% probability displacement ellipsoids for non-H atoms.

**Fig. 2.**
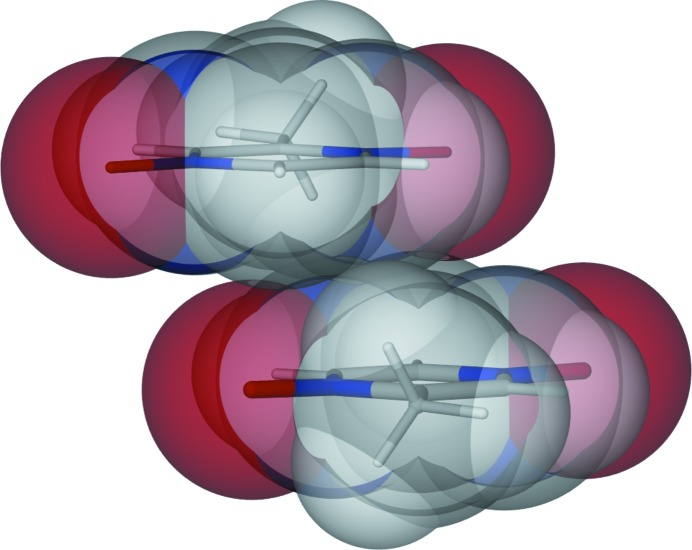
π-π interactions between neighboring 2-methylpyrazine 1,4-dioxide molecules.

**Fig. 3.**
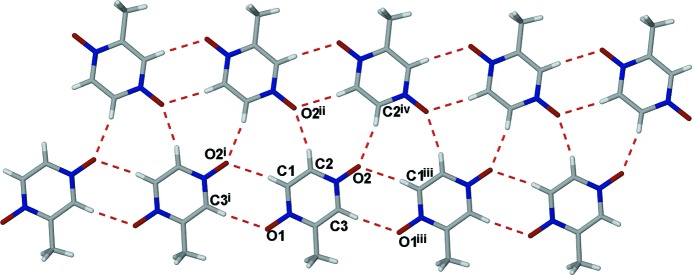
C—H···O hydrogen bonding interactions between neighboring 2-methylpyrazine 1,4-dioxide molecules. Hydrogen bonds are shown as dashed lines. Symmetry codes: (i) x - 1, y, z; (ii) x - 1/2, y, -z + 3/2; (iii) x + 1, y, z; (iv) x + 1/2, y, -z + 3/2.

**Fig. 4.**
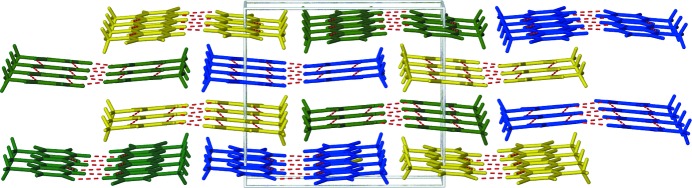
Packing of the title compound viewed down the a axis. Color scheme indicates individual C—H···O hydrogen bonded ribbons. Hydrogen bonds are shown as dashed lines

## supplementary crystallographic information

### Comment

The use of pyrazine *N,N*'-dioxide in the synthesis of lanthanide coordination networks has been of recent interest (Cardoso *et al.* (2001), Sun *et al.* (2004)). Shi *et al.* (2006) recently reported the use 2-methylpyrazine 1,4-dioxide in the synthesis of a cadmium (II) coordination network, and Sun *et al.* (2005), Xu *et al.* (2005*a*), and Xu *et al.* (2005*b*) report its use in the synthesis of several molecular complexes. The title compound was prepared using the reaction the conditions described by Simpson *et al.* (1963) to prepare 2,2'-bipyridine *N,N*'-dioxide.

The asymmetric unit of the title compound contains one 2-methylpyrazine 1,4-dioxide molecule (Figure 1). π-π stacking interactions with a centroid to centroid distance of 3.7370 Å, an interplanar distance of 3.167 Å, and a slippage of 1.984 Å. are observed between neighboring N-oxide molecules [symmetry code: -*x* + 1, -*y* + 1, -*z* + 1] (Figure 2). The title compound forms six C—H···O hydrogen bonds with four neighboring N-oxide molecules, and these hydrogen bonding interactions result in the formation of one-dimensional ribbons that propagate parallel to the *a* axis (Figure 4). As seen in the packing diagram, each one-dimensional ribbon is surrounded by six similar ribbons. (Figure 5)

### Experimental

2-Methylpyrazine (5.871 ml, 64.0 mmol), acetic acid (75 ml), and 30% hydrogen peroxide (13 ml) were heated at 343–353 K for 3 h. Additional hydrogen peroxide (9 ml) was added, and heating was continued. After an additional 19 h of heating the solution was cooled to room temperature. Crystals formed upon the addition of acetone (1*L*) and cooling to 273 K, and were recrystallized from hot water by addition of excess acetone and cooling to 273 K.

### Refinement

All H atoms were positioned geometrically and refined using a riding model with C—H = 0.95–0.99 Å and with *U_iso_*(H) = 1.2 (1.5 for methyl groups) times *U*_eq_(C).

### Crystal data

**Table:**

C_5_H_6_N_2_O_2_	*F*(000) = 528
*M**_r_* = 126.12	*D*_x_ = 1.566 Mg m^−^^3^
Orthorhombic, *P**b**c**a*	Mo *K*α radiation, λ = 0.71073 Å
Hall symbol: -P 2ac 2ab	Cell parameters from 1890 reflections
*a* = 6.3953 (9) Å	θ = 3.0–31.5°
*b* = 12.2472 (18) Å	µ = 0.12 mm^−^^1^
*c* = 13.6613 (19) Å	*T* = 173 K
*V* = 1070.0 (3) Å^3^	Rod, colorless
*Z* = 8	0.53 × 0.20 × 0.15 mm

### Data collection

**Table:**

Bruker SMART APEX CCD diffractometer	1708 independent reflections
Radiation source: fine-focus sealed tube	1407 reflections with *I* > 2σ(*I*)
graphite	*R*_int_ = 0.025
ω scans	θ_max_ = 31.8°, θ_min_ = 3.0°
Absorption correction: multi-scan (*SADABS*; Bruker, 2001)	*h* = −9→8
*T*_min_ = 0.763, *T*_max_ = 1.000	*k* = −17→8
5556 measured reflections	*l* = −11→19

### Refinement

**Table:**

Refinement on *F*^2^	Primary atom site location: structure-invariant direct methods
Least-squares matrix: full	Secondary atom site location: difference Fourier map
*R*[*F*^2^ > 2σ(*F*^2^)] = 0.046	Hydrogen site location: inferred from neighbouring sites
*wR*(*F*^2^) = 0.122	H-atom parameters constrained
*S* = 1.07	*w* = 1/[σ^2^(*F*_o_^2^) + (0.0537*P*)^2^ + 0.6359*P*] where *P* = (*F*_o_^2^ + 2*F*_c_^2^)/3
1708 reflections	(Δ/σ)_max_ < 0.001
83 parameters	Δρ_max_ = 0.45 e Å^−^^3^
0 restraints	Δρ_min_ = −0.31 e Å^−^^3^

### Special details

**Table:**

Geometry. All e.s.d.'s (except the e.s.d. in the dihedral angle between two l.s. planes) are estimated using the full covariance matrix. The cell e.s.d.'s are taken into account individually in the estimation of e.s.d.'s in distances, angles and torsion angles; correlations between e.s.d.'s in cell parameters are only used when they are defined by crystal symmetry. An approximate (isotropic) treatment of cell e.s.d.'s is used for estimating e.s.d.'s involving l.s. planes.
Refinement. Refinement of *F*^2^ against ALL reflections. The weighted *R*-factor *wR* and goodness of fit *S* are based on *F*^2^, conventional *R*-factors *R* are based on *F*, with *F* set to zero for negative *F*^2^. The threshold expression of *F*^2^ > σ(*F*^2^) is used only for calculating *R*-factors(gt) *etc*. and is not relevant to the choice of reflections for refinement. *R*-factors based on *F*^2^ are statistically about twice as large as those based on *F*, and *R*- factors based on ALL data will be even larger.

### Fractional atomic coordinates and isotropic or equivalent isotropic displacement parameters (Å^2^)

**Table:**

	*x*	*y*	*z*	*U*_iso_*/*U*_eq_	
O1	0.33101 (13)	0.64269 (8)	0.41676 (7)	0.0164 (2)	
O2	0.99921 (14)	0.59296 (8)	0.64921 (7)	0.0183 (2)	
N1	0.49403 (15)	0.63273 (8)	0.47271 (8)	0.0122 (2)	
N2	0.83721 (15)	0.60672 (9)	0.59294 (8)	0.0128 (2)	
C1	0.47150 (19)	0.62449 (10)	0.57203 (9)	0.0136 (2)	
H1	0.3386	0.6279	0.5995	0.016*	
C2	0.64141 (19)	0.61132 (10)	0.63137 (9)	0.0142 (2)	
H2	0.6230	0.6055	0.6987	0.017*	
C3	0.86033 (18)	0.61629 (10)	0.49464 (9)	0.0126 (2)	
H3	0.9940	0.6141	0.4679	0.015*	
C4	0.69092 (18)	0.62919 (10)	0.43327 (9)	0.0125 (2)	
C5	0.7106 (2)	0.63969 (11)	0.32565 (9)	0.0165 (2)	
H5A	0.6439	0.7059	0.3046	0.025*	
H5B	0.6448	0.5783	0.2946	0.025*	
H5C	0.8559	0.6417	0.3080	0.025*	

### Atomic displacement parameters (Å^2^)

**Table:**

	*U*^11^	*U*^22^	*U*^33^	*U*^12^	*U*^13^	*U*^23^
O1	0.0092 (4)	0.0223 (5)	0.0177 (4)	0.0005 (3)	−0.0044 (3)	0.0008 (3)
O2	0.0112 (4)	0.0298 (5)	0.0140 (4)	0.0004 (3)	−0.0053 (3)	0.0002 (4)
N1	0.0090 (4)	0.0139 (4)	0.0138 (5)	0.0001 (3)	−0.0009 (4)	−0.0004 (3)
N2	0.0103 (4)	0.0169 (5)	0.0113 (5)	−0.0005 (3)	−0.0013 (4)	−0.0003 (4)
C1	0.0118 (5)	0.0155 (5)	0.0136 (5)	0.0000 (4)	0.0027 (4)	0.0001 (4)
C2	0.0137 (5)	0.0167 (5)	0.0122 (5)	−0.0006 (4)	0.0026 (4)	−0.0006 (4)
C3	0.0097 (4)	0.0160 (5)	0.0120 (5)	−0.0007 (4)	0.0013 (4)	−0.0006 (4)
C4	0.0104 (5)	0.0143 (5)	0.0126 (5)	0.0000 (4)	0.0008 (4)	−0.0004 (4)
C5	0.0142 (5)	0.0242 (6)	0.0112 (5)	0.0008 (4)	0.0007 (4)	0.0015 (5)

### Geometric parameters (Å, °)

**Table:**

O1—N1	1.2985 (13)	C2—H2	0.9300
O2—N2	1.3011 (13)	C3—C4	1.3789 (16)
N1—C1	1.3681 (16)	C3—H3	0.9300
N1—C4	1.3703 (15)	C4—C5	1.4813 (18)
N2—C3	1.3561 (16)	C5—H5A	0.9600
N2—C2	1.3590 (15)	C5—H5B	0.9600
C1—C2	1.3653 (17)	C5—H5C	0.9600
C1—H1	0.9300		
			
O1—N1—C1	120.41 (10)	N2—C3—C4	121.75 (11)
O1—N1—C4	120.62 (10)	N2—C3—H3	119.1
C1—N1—C4	118.97 (10)	C4—C3—H3	119.1
O2—N2—C3	120.63 (10)	N1—C4—C3	119.11 (11)
O2—N2—C2	120.72 (10)	N1—C4—C5	117.75 (11)
C3—N2—C2	118.65 (10)	C3—C4—C5	123.14 (11)
C2—C1—N1	120.92 (11)	C4—C5—H5A	109.5
C2—C1—H1	119.5	C4—C5—H5B	109.5
N1—C1—H1	119.5	H5A—C5—H5B	109.5
N2—C2—C1	120.59 (11)	C4—C5—H5C	109.5
N2—C2—H2	119.7	H5A—C5—H5C	109.5
C1—C2—H2	119.7	H5B—C5—H5C	109.5

### Hydrogen-bond geometry (Å, °)

**Table:**

*D*—H···*A*	*D*—H	H···*A*	*D*···*A*	*D*—H···*A*
C1—H1···O2^i^	0.93	2.31	3.2224 (16)	165
C2—H2···O2^ii^	0.93	2.23	3.1405 (17)	167
C3—H3···O1^iii^	0.93	2.29	3.2090 (15)	168

Symmetry codes: (i) *x*−1, *y*, *z*; (ii) *x*−1/2, *y*, −*z*+3/2; (iii) *x*+1, *y*, *z*.

## References

[bb1] Barbour, L. J. (2001). *J. Supramol. Chem* **1**, 189-191.

[bb2] Bruker (2001). *SADABS.* Bruker AXS Inc., Madison, Wisconsin, USA.

[bb3] Bruker (2007). *SMART* and *SAINT-Plus*. Bruker AXS Inc., Madison, Wisconsin, USA.

[bb4] Cardoso, M. C. C., Zinner, L. B., Zukerman-Scheptor, J., Araújo Melo, D. M. & Vincentini, G. J. (2001). *J. Alloys Compd*, **323–324**, 22–25.

[bb5] Sheldrick, G. M. (2008). *Acta Cryst.* A**64**, 112–122. 10.1107/S0108767307043930 18156677

[bb6] Shi, J. M., Zhang, F. X., Zhang, X., Xu, H. Y., Lui, L. D. & Ma, J. P. (2006). *Chin. J. Struct. Chem.* **20**, 1238–1242.

[bb7] Simpson, P. G., Vinciguerra, A. & Quagliano, J. V. (1963). *Inorg. Chem.* **2**, 282–286.

[bb8] Sun, H. L., Gao, S., Ma, B. Q., Chang, F. & Fu, W. F. (2004). *Microporous Mesoporous Mater.* **73**, 89–95.

[bb9] Sun, Y.-M., Shi, J.-M. & Zhang, X. (2005). *Acta Cryst.* E**61**, m1501–m1502.

[bb10] Xu, W., Shi, J. M. & Zhang, X. (2005*a*). *Acta Cryst.* E**61**, m854–m855.

[bb11] Xu, W., Shi, J.-M. & Zhang, X. (2005*b*). *Acta Cryst.* E**61**, m2194–m2195.

